# Lightweight Transmission Line Fault Detection Method Based on Leaner YOLOv7-Tiny

**DOI:** 10.3390/s24020565

**Published:** 2024-01-16

**Authors:** Qingyan Wang, Zhen Zhang, Qingguo Chen, Junping Zhang, Shouqiang Kang

**Affiliations:** 1 School of Measurement-Control and Communication Engineering, Harbin University of Science and Technology, Harbin 150080, China; wangqy@hrbust.edu.cn (Q.W.); 2120610167@stu.hrbust.edu.cn (Z.Z.); kangshouqiang@hrbust.edu.cn (S.K.); 2 School of Electronics and Information Engineering, Harbin Institute of Technology, Harbin 150001, China; zhangjp@hit.edu.cn

**Keywords:** fault detection, Leaner YOLOv7-Tiny, lightweight, transmission lines, YOLOv7-Tiny

## Abstract

Aiming to address the issues of parameter complexity and high computational load in existing fault detection algorithms for transmission lines, which hinder their deployment on devices like drones, this study proposes a novel lightweight model called Leaner YOLOv7-Tiny. The primary goal is to swiftly and accurately detect typical faults in transmission lines from aerial images. This algorithm inherits the ELAN structure from YOLOv7-Tiny network and replaces its backbone with depthwise separable convolutions to reduce model parameters. By integrating the SP attention mechanism, it fuses multi-scale information, capturing features across various scales to enhance small target recognition. Finally, an improved FCIoU Loss function is introduced to balance the contribution of high-quality and low-quality samples to the loss function, expediting model convergence and boosting detection accuracy. Experimental results demonstrate a 20% reduction in model size compared to the original YOLOv7-Tiny algorithm. Detection accuracy for small targets surpasses that of current mainstream lightweight object detection algorithms. This approach holds practical significance for transmission line fault detection.

## 1. Introduction

Target detection technology, evolving with time, is now extensively applied in transmission line fault detection. Currently, this application falls into two main categories. The first combines image processing technology with machine learning. The second relies on deep learning models. Each method has distinct characteristics and plays a crucial role in identifying faults in transmission lines.

Image processing and machine learning-based detection algorithms for transmission lines involve three core components. The first step is identifying the target region [1]. This is crucial, as aerial images often include complex backgrounds like mountains and buildings. To mitigate external influences on detection, preprocessing is essential for isolating the target region. This focus on the target area enables effective feature extraction, minimizing interference from external factors and enhancing detection accuracy.

Next, features are manually generated from the target region. These include color attributes, morphological features, scale-invariant feature transformations [2], and directional gradient histogram features [3]. Finally, machine learning algorithms, such as support vector machines [4], decision trees, and AdaBoost classifiers [5], use these crafted feature vectors for training and classification. This approach underpins the reliability and precision of target detection in transmission lines.

Recently, scholars have increasingly employed image processing and machine learning classifiers for detecting faults in transmission lines. For instance, Ref. [6] introduced a method using sparse representation algorithms to identify insulator faults. This technique initially employs the Hough transform for detecting straight lines, aiding in insulator localization. Subsequent classification via SVM classifiers refines this positioning. Further, an overcomplete dictionary for sparse representation classifiers is constructed, utilizing feature vectors from cracked and dropped string insulators for accurate fault identification.

Another study, Ref. [7], utilized Local Binary Pattern (LBP) and Histogram of Oriented Gradients (HOG) techniques for extracting local features of insulators. Machine learning algorithms then train classifiers for precise insulator identification, focusing on crack analysis. This study also compared four feature matching techniques: Affine-SIFT (ASIFT), Speeded Up Robust Features (SURF), Oriented Fast and Rotated Brief (ORB), and Fast Retina Keypoint (FREAK), targeting accurate detection of components like locating supports.

In addition, Ref. [8] explored insulator feature extraction using discrete orthogonal S-transform. These features were then used to train SVM classifiers, enhancing the detection of insulator faults.

Recent advances in deep learning have led to its widespread application across various fields, particularly impacting target detection. Deep learning models in target detection adopt an innovative approach, eliminating the need for manually crafted features. Instead, they actively acquire and understand various deep features of insulators and potential faults through full, semi, and unsupervised learning methods. This iterative training enhances the models’ robustness and generalizability.

For instance, Ref. [9] demonstrates improved detection accuracy for small target insulators by incorporating residual structures into Faster R-CNN [10] and introducing an enhanced feature pyramid. Similarly, in Ref. [11], detection accuracy of small target insulator damage was enhanced through the incorporation of residual structures into MASK R-CNN, coupled with the introduction of an improved attention mechanism.

In contrast to these two-stage target detection algorithms, the most widely used and rapidly evolving are the YOLO series, introduced by Joseph et al. [12] in 2015. These single-stage detection networks are simple yet efficient, employing an end-to-end training approach. This methodology simplifies the model’s design and training process, avoiding the complexities of multi-stage optimization.

The YOLO series has evolved swiftly, with the introduction of YOLOv3 [13], YOLOv4 [14], and YOLOv5. Ref. [15] enhances YOLOv5 by adding attention mechanisms and incorporating a receptive field module. This extract features at various scales, improving detection accuracy. Following the development of YOLOv5 by the Ultralytics team in 2020, the YOLOv4 developers introduced YOLOv7 [16] within just two years. YOLOv7 significantly surpasses YOLOv5 in detection speed and accuracy. Ref. [17] improves YOLOv7 further by integrating attention mechanisms and a novel Intersection over Union (IoU) Loss function, thus enhancing the algorithm’s detection precision.

However, these improvements in the YOLO algorithm’s detection accuracy come at a cost of increased parameters and computational load. This escalates hardware requirements for drone deployment. To address these challenges, this paper introduces Leaner YOLOv7-Tiny, a streamlined model for transmission line fault detection based on YOLOv7-Tiny. Leaner YOLOv7-Tiny effectively reduces the model’s parameters while boosting accuracy in detecting small targets. This optimization not only fits drone deployment criteria but also enhances target detection precision. The contributions of this paper are summarized as follows:

(1) Maintaining the Efficient Layer Aggregation Networks (ELAN) structure of the YOLOv7-Tiny network, this approach substitutes the backbone’s standard convolution with depth-separable convolution from the PP-LCNet [18] network. This change splits the ordinary convolution into depth and point-by-point convolutions, significantly reducing the parameter count.
(2) Building on spatial attention, the SP attention mechanism introduces convolutional kernels of varied sizes. This enhancement enables multi-scale feature extraction, bolstering the model’s proficiency in detecting small targets while preserving its lightweight nature.
(3) The introduction of an improved FCIoU Loss function strategically balances the impact of high-quality and low-quality samples on the Loss. This advancement accelerates model convergence and enhances detection accuracy.

In a pioneering approach, this paper enhances the YOLOv7-Tiny network’s ELAN structure by substituting standard convolution with depth-separable convolution from the PP-LCNet, effectively reducing the parameter count. Introducing the SP attention mechanism, convolutional kernels of varied sizes enable multi-scale feature extraction, elevating the model’s capability to detect small targets while maintaining a lightweight profile. The innovation extends to an improved FCIoU Loss function, strategically balancing the impact of high- and low-quality samples, accelerating model convergence, and enhancing detection accuracy. These novel adaptations collectively underscore the study’s commitment to advancing both efficiency and performance in object detection.

The paper is structured as follows: Section 2 delves into the theoretical aspects of YOLOv7-Tiny. Section 3 details each innovative module. Section 4 presents the dataset, evaluates the proposed method’s performance, and compares it with current mainstream lightweight networks. The conclusion is provided in Section 5.

## 2. Basics of YOLOv7-Tiny Algorithm

YOLOv7, a leading object detection algorithm, excels in speed and accuracy, with performance ranging from 5 FPS to 160 FPS. It offers various model sizes, including YOLOv7-Tiny, YOLOv7, YOLOvX, and YOLOvW. This study focuses on model lightweightness, selecting YOLOv7-Tiny as the foundational model.

YOLOv7-Tiny, compared to YOLOv5s, incorporates the ELAN architecture for feature extraction. ELAN elevates the base network’s learning capacity by expanding, transforming, and aggregating features. It also accelerates model convergence through controlled gradient pathways. The use of group convolution expands the channels of computational blocks, maintaining the transformation layers’ structure. This process enhances the backbone network’s feature-learning capability and optimizes parameter utilization in computations.

The Merge and Process (MP) module in the network is bifurcated into two distinct branches. The first branch is designed for downsampling, utilizing max-pooling followed by a 1 × 1 convolutional layer for channel adjustment. The second branch, on the other hand, modifies channel numbers through a 1 × 1 convolutional layer, employs a 3 × 3 convolutional kernel, and uses a convolution operation with a stride of 2 for downsampling. The outputs from these branches are then merged, resulting in a super-downsampled output that significantly boosts the backbone network’s feature extraction efficiency.

**Figure 1:**
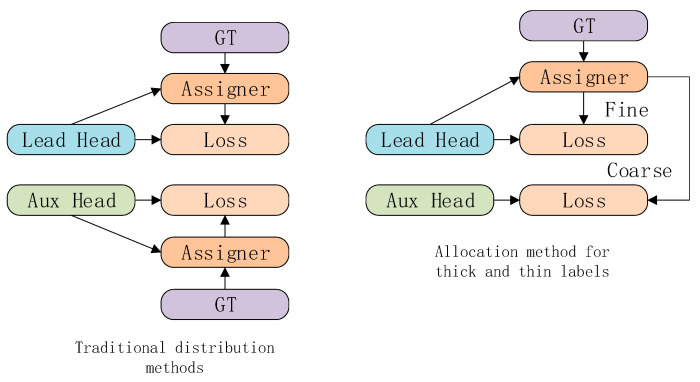
Comparison Diagram of Deep Supervision Frameworks.

In the Head section, the model employs deep supervision techniques, a departure from conventional methods. It introduces additional auxiliary heads to boost multi-task object detection performance. This novel strategy diverges from the traditional separation of auxiliary and guiding heads. Instead, it uses the guiding head to create hierarchical labels, ranging from coarse to fine. These labels are then individually utilized for training by both the auxiliary and guiding heads, enhancing learning efficiency. This concept is visually represented in Figure 1.

The guiding head plays a pivotal role in this model. It directs the label allocator and the predicted Ground Truth (GT) in a computational process. Through optimization, it generates a set of soft labels. These soft labels then become the training targets for both the auxiliary and guiding heads. The aim is to fortify the guiding head’s learning capabilities. This enhancement allows the soft labels to more accurately reflect the distributional nuances and relevance between the source data and the targets.

The model’s learning approach resembles generalized residual learning. It permits the shallower auxiliary head to directly assimilate information processed by the guiding head. Consequently, the guiding head concentrates on unlearned residual information. In this setup, fine labels correspond to the soft labels from the guiding head’s label allocator. Coarse labels emerge by broadening the allocation of positive samples to include more grid cells as positive targets.

## 3. The Proposal of the Leaner YOLOv7-Tiny Algorithm

YOLOv7-Tiny, a compact variant in the YOLOv7 series, offers scope for enhancements in its loss function and small object detection accuracy. Building on this, the paper introduces Leaner YOLOv7-Tiny, a more streamlined and efficient algorithm. Leaner YOLOv7-Tiny retains the ELAN structure and auxiliary training approach from YOLOv7-Tiny, but modifies the backbone network with depthwise separable convolutions to minimize model size. Additionally, it incorporates the SP multi-scale spatial attention mechanism for improved target feature extraction and adopts FCIoU Loss to quicken model convergence and boost detection accuracy.

**Figure 2:**
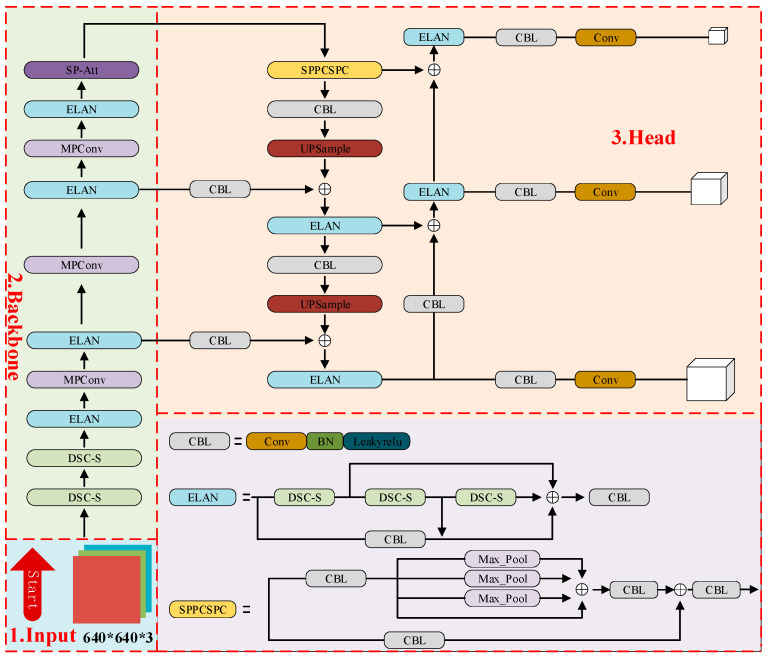
Structure Diagram of Leaner YOLOv7-Tiny.

Figure 2 illustrates the processing of a 640 × 640 × 3 RGB image in Leaner YOLOv7-Tiny. Initially, the image is processed through two rounds of depthwise separable convolutions, leading into the ELAN module for efficient feature aggregation. This is followed by three combined structures of MPConv and ELAN. MPConv functions through dual branches, merging their outcomes for super-downsampling. The process then transitions to the SP module, which captures varied receptive fields, thus enhancing the comprehension and processing of multi-scale features. The final output consists of three feature map sets: 80 × 80 × 128, 40 × 40 × 256, and 20 × 20 × 512, at the neck output.

### 3.1. DepthSepConv-S Depthwise Separable Network

PP-LCNet, a CPU-optimized lightweight network, outperforms popular counterparts like MobileNetV3 [19] and ShuffleNetV2 [20] in terms of parameters and accuracy. In this study, YOLOv7-Tiny’s ELAN module is refined by replacing its standard Conv convolution with PP-LCNet’s DepthSepConv. Additionally, the ReLU activation function is substituted with SiLU, culminating in a new convolution variant termed DepthSepConv-S.

DepthSepConv convolution comprises three key components: depthwise convolution, a squeeze-and-excitation (SE) layer, and pointwise convolution. In depthwise convolution, each channel of the input feature map is processed independently. A convolution kernel convolves each channel to produce an intermediate feature map with an identical channel count. This process solely targets the depth dimension of the input feature map, excluding inter-channel interactions. Depthwise convolution notably diminishes parameter count and computational complexity.

**Figure 3:**
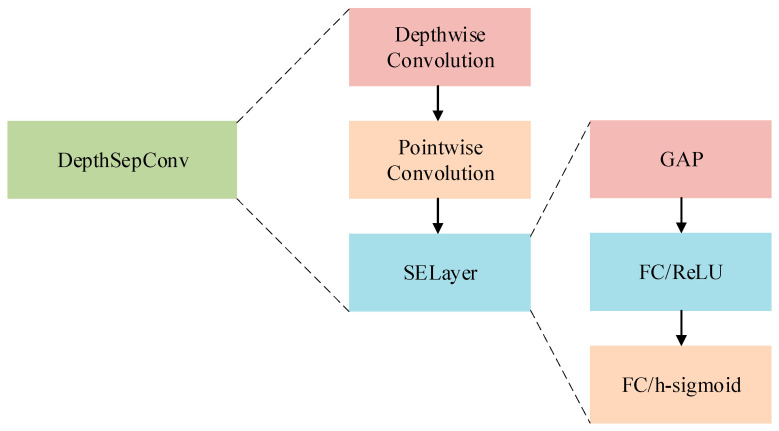
Structure Diagram of DepthSepConv.

Depthwise separable convolution, a fusion of depthwise and pointwise convolutions, markedly cuts down on parameters and computational complexity. Depthwise convolution specifically addresses the depth dimension of the feature map, whereas pointwise convolution integrates inter-channel features. This strategic separation boosts the model’s capabilities in learning and expressing features while ensuring computational efficiency. Ideal for lightweight models and constrained settings like unmanned aerial vehicles (UAVs), its utility is visually depicted in Figure 3.

The DepthSepConv layer depicted in the diagram manifests as a dual-stage process, comprising a depthwise convolution succeeded by a pointwise convolution. Notably, this architectural configuration incorporates a Squeeze-and-Excitation (SE) layer, intricately recalibrating channel-wise feature responses to explicitly model interdependencies among channels. Complementing this, the presence of a Global Average Pooling (GAP) layer and fully connected layers, activated by ReLU and sigmoid functions, underscores the architectural focus on refining feature extraction and optimizing utility for classification tasks within the network. This design epitomizes a meticulous integration of depthwise and pointwise convolutions, coupled with attention mechanisms, to enhance the network’s capacity for sophisticated feature processing and classification.

The computational cost for executing a convolution operation on images, using kernels of size *K* and *M* channels to yield an output feature map with *N* channels, is quantifiable, as illustrated in Equation (1). In this equation, $D_{K}$ represents the size of the depth convolution kernel. FLOPs represent the amount of computation. *M* and *N* are the number of input and output channels, respectively.

(1) $$\mathrm{FLOPs}=D_{K}\times D_{K}\times M\times N$$

when employing depthwise separable convolution operations; the associated computational cost is outlined in Equation (2).

(2) $$\mathrm{FLOPs}=D_{K}\times D_{K}\times M+1\times 1\times M\times N$$

Equation (3) demonstrates that using depthwise separable convolution for feature extraction notably lowers computational load compared to standard convolution.

(3) $$\frac{D_{K}\times D_{K}\times M+1\times 1\times M\times N}{D_{K}\times D_{K}\times M\times N}=\frac{1}{D_{K}^{2}}+\frac{1}{N}$$

Originally, the DepthSepConv positioned the SE layer post point-wise convolution, hindering its ability to effectively capture channel-specific features. To remedy this, a reconfiguration is proposed: shifting the SE layer to follow depthwise convolution instead. This adjustment grants the SE layer direct access for adaptive feature selection per channel. Such a modification refines channel weight control, enabling the network to more aptly align with the nuances of specific tasks and datasets. Consequently, this enhances the model’s expressiveness and overall performance.

Constrained by parameters and computational resources, deepening a model can foster the learning of more hierarchical abstract features. This depth enhances the model’s expressiveness and its proficiency in capturing intricate details and contextual information, thereby boosting accuracy. However, deeper networks often encounter challenges like gradient vanishing or exploding. These issues can impede model convergence or destabilize the training process.

**Figure 4:**
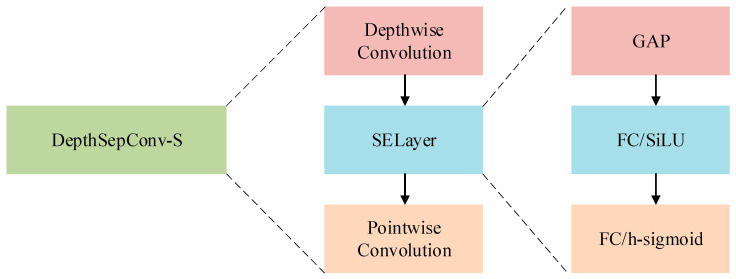
Structure Diagram of DepthSepConv-S.

To mitigate these challenges, DepthSepConv’s ReLU activation function is substituted with SiLU. SiLU’s smoother non-linear transformations help in addressing the gradient vanishing issue, preserving more input information. This shift promises enhanced model convergence and performance. Building on these modifications, the advanced DepthSepConv-S network emerges, aiming to counteract the gradient vanishing in deep networks and further refine detection accuracy. The structure of this network is illustrated in Figure 4.

The enhanced DepthSepConv-S network is applied to optimize channel-specific feature extraction, elevating overall expressiveness and model performance. Repositioning the SE layer post-depthwise convolution addresses previous limitations, enabling adaptive feature recalibration and improved channel weight control, crucial for mitigating issues like gradient vanishing in deep networks. Substituting ReLU with SiLU in DepthSepConv-S enhances non-linear transformations, mitigating convergence challenges and preserving input information. These strategic modifications promote refined model convergence, performance, and detection accuracy, enabling the network to adeptly learn hierarchical abstract features and handle diverse tasks and datasets with increased efficacy.

### 3.2. SP Multi-Scale Spatial Attention Mechanism

SENet [21], CBAM [22], and GAM [23] are prominent attention mechanisms in deep learning. SENet boosts network expressiveness by introducing channel attention, highlighting significant feature channels. CBAM merges channel and spatial attention, capturing feature correlations across both dimensions. GAM utilizes global attention to enhance global feature relationships, thereby improving feature representation.

CBAM and GAM attention mechanisms significantly improve model detection accuracy. However, CBAM, utilizing a single convolutional kernel, struggles to capture multi-scale features, particularly in small object detection. Conversely, GAM processes the entire feature map, resulting in a high parameter count. Each convolution in GAM adds learnable parameters, as it comprises multiple layers to understand feature relationships. Despite GAM’s effectiveness, its substantial parameter size limits its suitability for lightweight models.

**Figure 5:**
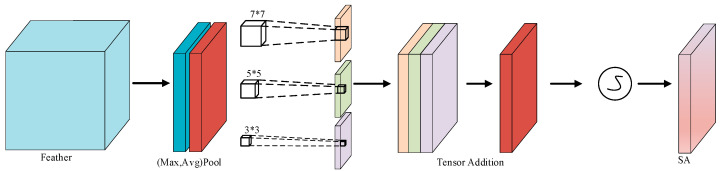
SP Multi-Scale Spatial Attention Mechanism.

Acknowledging the integration of the SENet channel attention mechanism in the DepthSepConv-S network, a novel multi-scale spatial attention mechanism, named SP attention, is proposed. This mechanism enhances the basic spatial attention concept by incorporating multi-scale convolutional kernels. Such a design enables the capture of diverse receptive fields, fostering a deeper understanding and processing of features across various scales. The architecture of this mechanism is illustrated in Figure 5.

The input tensor X, sized H × W × C, undergoes initial processing via max pooling and average pooling, creating max-pooled and average-pooled feature maps. These maps are then concatenated along the channel dimension, amalgamating features from both operations. Subsequently, the combined feature maps are convolved with 3 × 3, 5 × 5, and 7 × 7 kernels to produce multi-scale features. The resulting features are concatenated, encompassing diverse scale attributes. This process is critical for small object detection, as these objects, due to their diminutive size, necessitate multi-scale feature analysis to effectively discern details and contextual information.

### 3.3. The FCIoU Loss Function

The Complete Intersection over Union (CIoU) loss function, incorporated in YOLOv7, represents an advancement over traditional loss functions. It aims to more precisely quantify the distance and similarity between bounding boxes. CIoU introduces additional terms for distance and aspect ratio, extending beyond basic *IoU* to thoroughly assess the similarities and differences between bounding boxes. This concept is encapsulated in Equation (4).

(4) $$L_{CIoU}=1-IoU+\frac{\rho^{2}(b,b^{gt})}{c^{2}}+\alpha \upsilon$$

In the Equation, b and $b^{gt}$ represent the centroids of the target box and the predicted box, respectively. ρ denotes the Euclidean distance between these two points. α is a parameter used for trade-off, while ν measures aspect ratio consistency. c represents the distance of the minimum enclosing rectangle diagonal.

The introduction of Focal and Efficient IoU Loss (Focal EIoU) [24] addresses two key issues in current loss functions. Firstly, it targets the inefficiency in guiding bounding box regression training; this inefficiency not only slows model convergence but also impacts regression accuracy. Secondly, it tackles the problem of unbalanced anchor box quality, which contributes to slower regression speeds in bounding box regression.

Power transmission lines, exposed to outdoor environments with complex backgrounds and harsh conditions, often result in varied image quality. During training, the predominance of low-quality samples can decelerate convergence due to their disproportionate influence on gradient contributions. To tackle this, FCIoU is introduced, an enhancement based on the Focal EIoU concept applied to CIoU. This advancement is detailed in Equation (5).

(5) $$L_{FCIoU}=CIoU^{\gamma }L_{CIoU}$$

**Figure 6:**
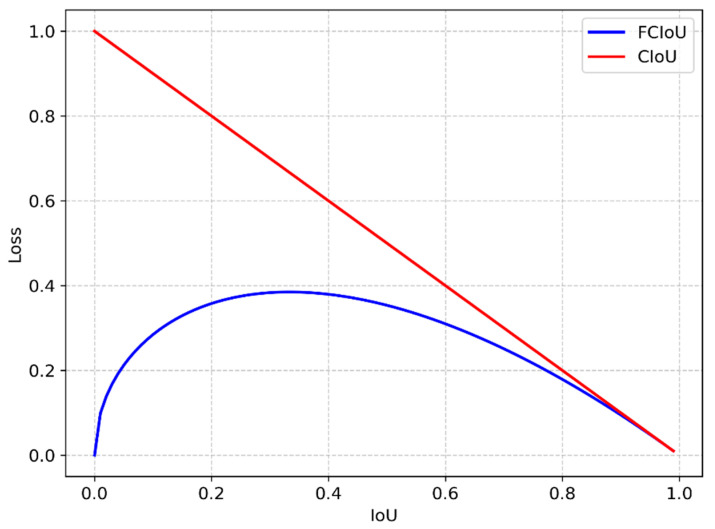
IoU Comparison Image.

In the equation, γ = 0.5. Multiplying the CIoU Loss by parameter $CIoU^{\gamma }$ aims to balance the contributions of high-quality and low-quality samples to the overall loss. It elevates the impact of high-quality samples (with larger IoU) while suppressing the contribution of low-quality samples (with smaller IoU), as illustrated in Figure 6.

In the referenced image, the IoU axis denotes IoU values, and the Loss axis represents the corresponding loss. The graph’s curves clearly demonstrate that FCIoU effectively lowers the loss from low-quality samples. This adjustment allows the network to concentrate more on high-quality samples, thereby expediting network convergence and ultimately improving the model’s detection accuracy.

## 4. Experiment and Analysis

To confirm the efficacy of the Leaner YOLOv7-Tiny algorithm, this section will conduct several comparative experiments. These tests will compare Leaner YOLOv7-Tiny against current mainstream lightweight networks, thoroughly validating its effectiveness.

### 4.1. Experimental Platform

The experimental setup used in this paper is as follows: the operating system employed was Ubuntu 18.04, running on an Intel(R) Xeon(R) Platinum 8255C CPU @ 2.50 GHz processor, with 40 GB of RAM. The GPU utilized was the NVIDIA GeForce RTX2080 Ti, PyTorch version 1.11.0, and CUDA version 11.3.

### 4.2. Dataset Description and Implementation Details

**Figure 7:**
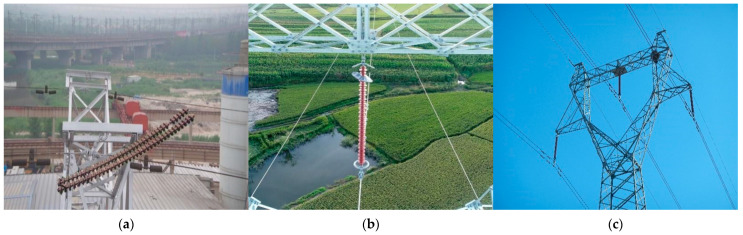
Example Diagram of Dataset. (**a**) Insulator Flashover. (**b**) Dropout of Equalizing Ring. (**c**) Bird’s Nest.

The dataset for this study comprises authentic images from unmanned aerial vehicles (UAVs), used in intelligent inspections by the State Grid Harbin Power Supply Company’s Inspection Center. It includes aerial photos of overhead transmission lines, frames from aerial videos, and publicly sourced insulator images from the internet. The dataset features various transmission line faults, such as insulator string bead explosion, missing equatorial ring, and bird nesting. Using the labelimg tool for annotation, we created label files. The definition of “Boom” corresponds to “insulator string bead explosion”, “Fall” represents “missing equatorial ring” on the insulator, and “Birds” indicates instances of bird nests. An illustration of the transmission line dataset is presented in Figure 7.

**Figure 8:**
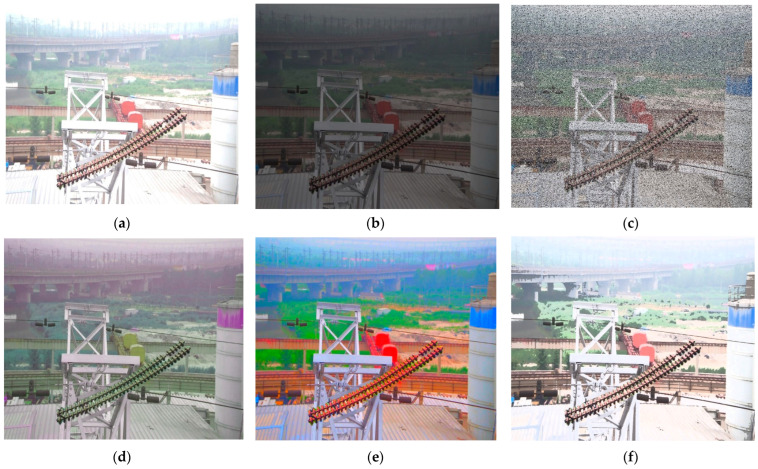
Six Example Images for Data Transformations. (**a**) Brightness Enhancement. (**b**) Reduced Brightness. (**c**) Noisy. (**d**) Color Shifted. (**e**) Color Enhanced. (**f**) Exposure.

The scarcity of diverse fault data in transmission lines necessitated image transformations to assess the Leaner YOLOv7-Tiny model’s generalization and robustness. Techniques like brightness enhancement, reduction, and noise addition were applied to dataset images. These methods simulate various outdoor lighting, weather conditions, and viewing angles. This comprehensive strategy effectively validates the proposed method’s efficacy. The impact of these data transformations is depicted in Figure 8.

The composition of the transformed dataset is depicted in Table 1 below. The dataset after transformations consists of a total of 3564 images, comprising 1855 samples of insulator flashover as Boom, 1090 samples of dropout of equalizing ring as Fall, and 619 samples of bird’s nests as Birds, representing the three typical fault categories.

### 4.3. Model Training Analysis

In the experiments, YOLOv7-Tiny served as the base network, and improvements were made using current mainstream lightweight models, including YOLOv7-Ghost, YOLOv7-Mobilenetv3, YOLOv7-Shffulenet, YOLOv7-EfficientLite, and Leaner YOLOv7-Tiny. These lightweight networks replaced the backbone network of YOLOv7-Tiny, with an initial learning rate set to 0.01. The maximum iteration count was set to 100, training on a dataset comprising 3000 images and testing on 564 images. Training was conducted in the same environment and on the same equipment to ensure the validity and consistency of the results.

**Figure 9:**
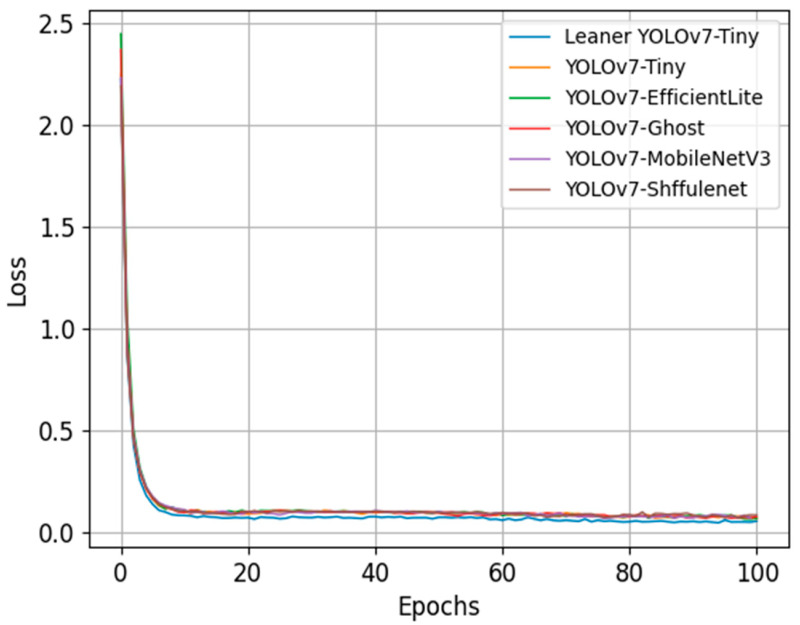
Loss Comparison Curves.

To clearly and accurately depict the changing trends of the loss function during the iterations of the six lightweight models, the loss curves during the iterative process of each algorithm were plotted using Matlab, as shown in Figure 9.

An examination of Figure 9 shows that the loss functions for all six algorithms demonstrate a decreasing trend, eventually converging to a specific threshold. Notably, the Leaner YOLOv7-Tiny lightweight detection algorithm, compared to the other five contenders, registers the lowest convergence value and exhibits the most stable loss variation. This pattern suggests that Leaner YOLOv7-Tiny is more efficient in error propagation along the gradient’s minimal path, effectively facilitating weight adjustments and updates.

**Figure 10:**
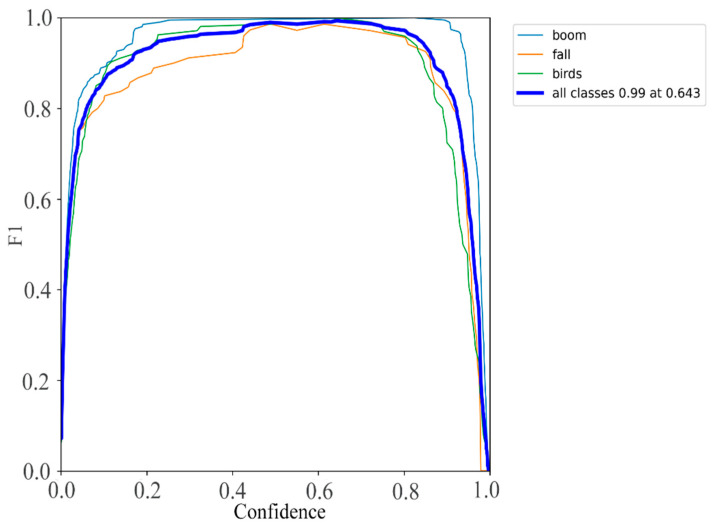
F1 Score Curve Chart.

Figure 10 presents the *F*1 curve of the Leaner YOLOv7-Tiny model. The *F*1 score, a harmonic mean of *precision* and *recall*, serves as a critical evaluation metric in machine learning, particularly for certain multi-class problems. Ranging between 0 and 1, as defined in Equation (6), *precision* represents the accuracy rate, and *recall* represents the *recall* rate. The *F*1 curve offers a comprehensive performance measure. Analysis of Figure 10 reveals that Leaner YOLOv7-Tiny consistently achieves high *F*1 scores, particularly within the confidence range of 0.6 to 0.7.

(6) $$F1=2\times \frac{precision\times recall}{precision+recall}$$

In Figure 10, the observed disparities in F1 curves across various categories can be attributed to inherent differences in sample distributions, feature characteristics, or the inherent complexity of each class. These variations reflect the nuanced performance of the model in handling diverse categories. A meticulous analysis of the shapes of individual class curves reveals potential fluctuations, shedding light on distinct challenges or complexities encountered by the model in classifying different categories. This nuanced understanding enhances our comprehension of the model’s discriminative capacity, offering insights into the specific intricacies associated with each class’s classification.

### 4.4. Evaluation Metrics

The evaluation of the object detection model in this paper is conducted using multiple metrics: mAP@0.5, mAP@0.5: 0.95, parameter count, model size, and Frames Per Second. mAP@0.5 is the mean average precision at an IoU threshold of 0.5, while mAP@0.5: 0.95 calculates the average precision over IoU thresholds ranging from 0.5 to 0.95, at 0.05 intervals. The parameter count quantifies the model’s learnable parameters, impacting its ability to fit training data and computational efficiency. Detection time measures the model’s speed in processing each image. Together, these metrics offer a thorough assessment of the model’s performance in various dimensions.

### 4.5. Comparative Experimental Analysis

This paper compares Leaner YOLOv7-Tiny with the current mainstream lightweight network detection models, as shown in Table 2 and Table 3. It can be observed that with the introduction of the DepthSepConv-S lightweight network, Leaner YOLOv7-Tiny reduces its parameters from 6.02 M in YOLOv7-Tiny to 4.67 M. While Leaner YOLOv7-Tiny is not the smallest model compared to other lightweight networks, it achieves higher accuracy, particularly in mAP@0.5: 0.95. This not only reflects Leaner YOLOv7-Tiny’s higher precision but also demonstrates the superior performance of the Leaner YOLOv7-Tiny network. Compared to YOLOv7-Mobilenetv3 and YOLOv7-Shffulenet, although Leaner YOLOv7-Tiny has a larger parameter count, it improves mAP@0.5 by 1% and 1.2%, respectively, and mAP@0.5: 0.95 by 6.1% and 6.8%. In contrast to YOLOv7-Ghost, YOLOv7-EfficientLite, and YOLOv7-Tiny, Leaner YOLOv7-Tiny has the fewest parameters and achieves the highest detection accuracy. This demonstrates that Leaner YOLOv7-Tiny effectively enhances the accuracy of model detection while maintaining a lightweight model size. As the model introduces an attention mechanism, Leaner YOLOv7-Tiny exhibits a slight increase in detection time compared to other lightweight models, yet still meets real-time requirements. In summary, the experiments confirm the effectiveness of the proposed Leaner YOLOv7-Tiny model in the task of detecting faults in transmission line scenarios.

**Figure 11:**
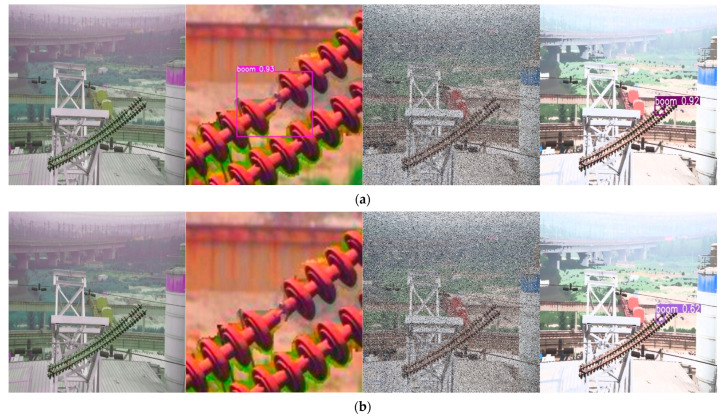

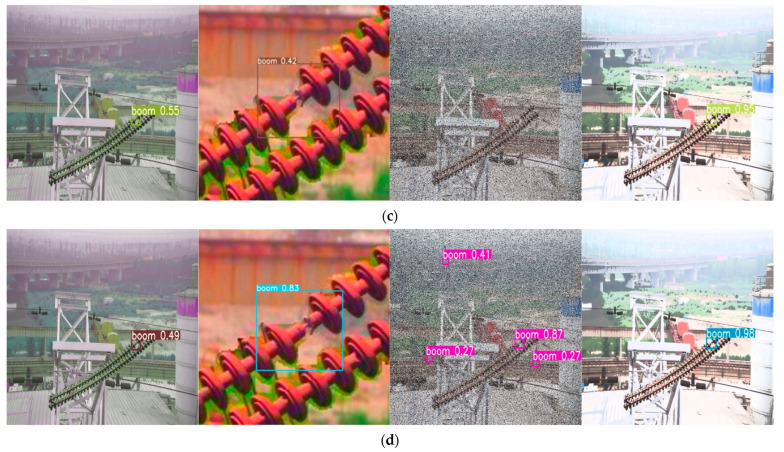

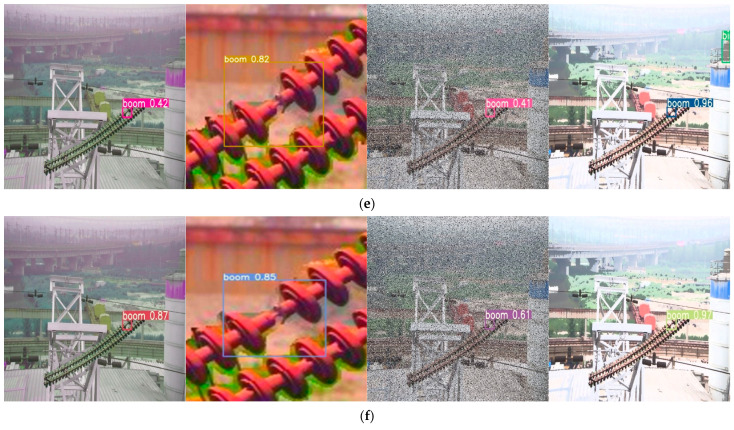
Comparison of Experimental Results. (**a**) YOLOv7-Mobilenetv3. (**b**) YOLOv7-Shffulenet. (**c**) YOLOv7-Ghost. (**d**) YOLOv7-EfficientLite. (**e**) YOLOv7-Tiny. (**f**) Leaner YOLOv7-Tiny.

Figure 11 details the results of comparative environment-based detection experiments, conducted to assess Leaner YOLOv7-Tiny’s robustness. These experiments revealed that mainstream lightweight models such as YOLOv7-Mobilenetv3, YOLOv7-Shffulenet, and YOLOv7-Ghost missed detections in various environments. While YOLOv7-EfficientLite and YOLOv7-Tiny identified all faults, they exhibited varying levels of false positives. In stark contrast, Leaner YOLOv7-Tiny consistently and accurately detected all faults, with no instances of misses or false detections. This performance highlights Leaner YOLOv7-Tiny’s robustness, demonstrating its capability to reliably detect faults in diverse, complex settings and effectively minimize errors like missing detections, false positives, and false negatives.

### 4.6. Ablation Experiments

Ablation experiments were performed to evaluate the contribution of each module in Leaner YOLOv7-Tiny, with the outcomes presented in Table 4. The introduction of a lightweight network initially led to a decrease in detection accuracy, attributed to the reduced parameter count. However, subsequent integration of the SP attention mechanism and FCIoU resulted in a gradual enhancement of accuracy. This improvement affirms the individual effectiveness of each module within Leaner YOLOv7-Tiny.

**Figure 12:**
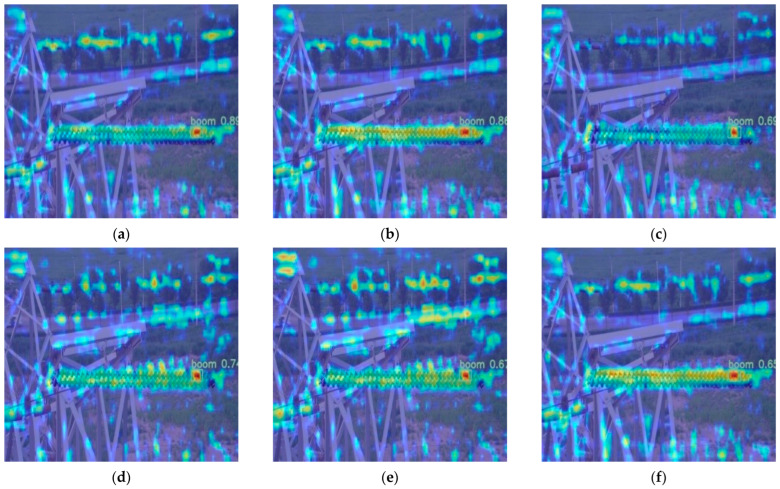
Attention Heatmaps. (**a**) Leaner YOLOv7-Tiny. (**b**) YOLOv7-Tiny. (**c**) YOLOv7-EfficientLite. (**d**) YOLOv7-Ghost. (**e**) YOLOv7-Shffulenet. (**f**) YOLOv7-Mobilenetv3.

For addressing the issue of small object detection, attention heatmaps were generated, as depicted in Figure 12. These are typically used to pinpoint discriminative regions for image classification and object detection, where stronger focus areas are highlighted in red. From the image, it is evident that compared to other lightweight models, the Leaner YOLOv7-Tiny used in this study exhibited a higher level of attention toward small objects, while other models were affected by background interference. This experiment confirms the effectiveness of Leaner YOLOv7-Tiny in small object detection.

## 5. Conclusions

Current transmission line fault detection algorithms have large parameters and high computational complexity, making UAV deployment difficult. Therefore, lightweight model improvements, while maintaining detection accuracy, are now a key research focus.

Drone-based transmission line inspections necessitate a lightweight model adept at precisely and swiftly detecting various faults in challenging natural environments. Responding to this need, this paper introduces a refined model based on YOLOv7-Tiny. This model, grounded in the YOLOv7-Tiny framework, offers an end-to-end solution for transmission line fault detection. It maintains YOLOv7-Tiny’s robust feature extraction while streamlining the workflow. Key advancements include integrating the DepthSepConv-S network and significantly reducing the model’s parameters and size for a lightweight design. Additionally, the SP spatial attention mechanism is introduced, merging multi-scale feature information to enhance detection accuracy, especially for small targets. Further refinement is achieved by upgrading the CIoU Loss to FCIoU, thereby sharpening the focus on high-quality samples and quickening network convergence, thus enhancing overall model detection accuracy.

The application of Depth Separable Networks from PP-LCNet to YOLOv7-Tiny led to a notable reduction in model size, a 20% decrease from 6.02 MB to 4.67 MB, which facilitates drone deployment. Ablation studies, however, indicated reductions in mAP@0.5 and mAP@0.5:0.95, attributed to decreased network parameters and computational requirements.

To counter the accuracy dip from the model’s lightweight nature, enhancements were introduced via the SP Multiscale Spatial Attention Mechanism. Employing convolutional kernels of varied sizes for feature extraction, this method aims to capture multi-scale features, thus heightening model detection accuracy and enhancing small target precision. For example, detection accuracy for ‘Boom’ and ‘Fall’ type faults improved significantly, demonstrating Leaner YOLOv7-Tiny’s enhanced precision compared to other lightweight networks.

The introduction of the improved FCIoU Loss function, modifying the existing CIoU Loss, addresses the inconsistency in aerial image quality. This enhancement effectively balances high- and low-quality sample contributions to the loss, speeding up model convergence and boosting detection accuracy.

In conclusion, the Leaner YOLOv7-Tiny model represents a significant leap forward in the realm of lightweight object detection, particularly for challenging applications like transmission line fault detection via drones. Its balanced approach to reducing model size while enhancing accuracy and robustness illustrates the potential of intelligent algorithms in real-world scenarios. The outcomes of this study not only contribute to the advancement of drone-based inspection technologies but also pave the way for future innovations in automated monitoring systems. As the field evolves, Leaner YOLOv7-Tiny will stand as a testament to the ongoing progress in optimizing performance within the constraints of resource-limited platforms.

## Figures and Tables

**Table 1:** Transmission Line Fault Dataset.

Categories	Before	After
Boom	372	1855
Fall	180	1090
Birds	130	619
All	682	3564

**Table 2:** Results of Model Performance Comparison.

Models	Para	Model Size	mAP@0.5	mAP@0.5: 0.95	Detection Time
YOLOv7-Mobilenetv3	4.17 M	8.3 MB	97.5%	63.4%	52 ms
YOLOv7-Shffulenet	4.3 M	8.5 MB	97.3%	62.7%	43 ms
YOLOv7-Ghost	6.27 M	12.4 MB	98.1%	68.3%	84 ms
YOLOv7-EfficientLite	6.39 M	12.6 MB	98.4%	67.8%	61 ms
YOLOv7-Tiny	6.02 M	11.7 MB	98.4%	69.3%	55 ms
Leaner YOLOv7-Tiny	4.67 M	9.2 MB	98.5%	69.5%	70 ms

**Table 3:** Fault Detection Performance Index.

Models	mAP@0.5			mAP@0.5: 0.95		
	Boom	Fall	Birds	Boom	Fall	Birds
YOLOv7-Mobilenetv3	98.4%	96.7%	97.9%	71.2%	59.4%	58.6%
YOLOv7-Shffulenet	98.4%	96.2%	98.3%	70.8%	59.1%	58.2%
YOLOv7-Ghost	98.5%	98%	97.8%	74.5%	64.6%	55.9%
YOLOv7-EfficientLite	98.5%	98.5%	98.1%	75.6%	64.2%	63.6%
YOLOv7-Tiny	98.5%	98%	98.4%	76.1%	68.8%	64.6%
Leaner YOLOv7-Tiny	98.5%	98.1%	98.3%	77.1%	69.3%	65.4%

**Table 4:** Ablation Experiment Results.

Models	mAP@0.5			mAP@0.5: 0.95		
	Boom	Fall	Birds	Boom	Fall	Birds
YOLOv7-tiny	98.5%	98%	98.4%	76.1%	68.8%	64.6%
Tiny+DepthSepConv-S	95.4%	95.3%	95.5%	74.8%	66.1%	62.4%
Tiny+DepthSepConv-S+SP	97.6%	97.4%	96.9%	77.5%	68.6%	64.4%
Leaner YOLOv7-Tiny	98.5%	98.1%	98.3%	77.1%	69.3%	65.4%

## Data Availability

Data are contained within the article.

## References

[1] Zhao Z.Q., Zheng P., Xu S.T., Wu X. (2019). Object detection with deep learning: A review. IEEE Trans. Neural Netw. Learn. Syst..

[2] Solis-Sánchez L.O., Castañeda-Miranda R., García-Escalante J.J., Torres-Pacheco I., Guevara-González R.G., Castañeda-Miranda C.L., Alaniz-Lumbreras P.D. (2011). Scale invariant feature approach for insect monitoring. Comput. Electron. Agric..

[3] Dalal N., Triggs B. (2005). Histograms of oriented gradients for human detection. Proceedings of the 2005 IEEE Computer Society Conference on Computer Vision and Pattern Recognition (CVPR’05).

[4] Chang C.C., Lin C.J. (2011). LIBSVM: A library for support vector machines. ACM Trans. Intell. Syst. Technol. TIST.

[5] Freund Y. (1995). Boosting a weak learning algorithm by majority. Inf. Comput..

[6] Li H. (2016). Research on Insulator Status Identification Method Based on Sparse Representation. Master’s Thesis.

[7] Yang H.J. (2017). Crack Detection and Positioning Bracket Inspection of Contact Network Insulators Based on Image Processing. Master’s Thesis.

[8] Reddy MJ B., Chandra B.K., Mohanta D.K. (2011). A DOST based approach for the condition monitoring of 11 kV distribution line insulators. IEEE Trans. Dielectr. Electr. Insul..

[9] Chen H.L. (2022). Research on Insulator Damage Detection Based on Improved Faster R-CNN Algorithm. Master’s Thesis.

[10] Ren S., He K., Girshick R., Sun J. (2015). Faster R-CNN: Towards real-time object detection with region proposal networks. Adv. Neural Inf. Process. Syst..

[11] Zhou M., Wang J., Li B. (2022). ARG-Mask RCNN: An Infrared Insulator Fault-Detection Network Based on Improved Mask RCNN. Sensors.

[12] Redmon J., Divvala S., Girshick R., Farhadi A. You only look once: Unified, real-time object detection. Proceedings of the IEEE Conference on Computer Vision and Pattern Recognition.

[13] Redmon J., Farhadi A. (2018). Yolov3: An incremental improvement. arXiv.

[14] Bochkovskiy A., Wang C.Y., Liao H.Y.M. (2020). Yolov4: Optimal speed and accuracy of object detection. arXiv.

[15] Hao S., Zhao X., Ma X., Zhang X., He T., Hou L. (2023). Multi-Class Defect Detection Method for Transmission Line Based on TR-YOLOv5. J. Graph..

[16] Wang C.Y., Bochkovskiy A., Liao H.Y.M. YOLOv7: Trainable bag-of-freebies sets new state-of-the-art for real-time object detectors. Proceedings of the IEEE/CVF Conference on Computer Vision and Pattern Recognition.

[17] Song Z.W., Huang X.B., Ji C., Zhang Y. (2023). Insulator Defect Detection and Fault Warning Method for Transmission Lines Based on Flexible YOLOv7. High Volt. Eng..

[18] Cui C., Gao T., Wei S., Du Y., Guo R., Dong S., Lu B., Zhou Y., Lv X., Liu Q. (2021). PP-LCNet: A lightweight CPU convolutional neural network. arXiv.

[19] Howard A., Sandler M., Chu G., Chen L.C., Chen B., Tan M., Wang W., Zhu Y., Pang R., Vasudevan V. Searching for mobilenetv3. Proceedings of the IEEE/CVF International Conference on Computer Vision.

[20] Ma N., Zhang X., Zheng H.T., Sun J. Shufflenet v2: Practical guidelines for efficient cnn architecture design. Proceedings of the European Conference on Computer Vision (ECCV).

[21] Tan M., Chen B., Pang R., Vasudevan V., Sandler M., Howard A., Le Q.V. Mnasnet: Platform-aware neural architecture search for mobile. Proceedings of the IEEE/CVF Conference on Computer Vision and Pattern Recognition.

[22] Woo S., Park J., Lee J.Y., Kweon I.S. Cbam: Convolutional block attention module. Proceedings of the European Conference on Computer Vision (ECCV).

[23] Liu Y., Shao Z., Hoffmann N. (2021). Global attention mechanism: Retain information to enhance channel-spatial interactions. arXiv.

[24] Zhang Y.F., Ren W., Zhang Z., Jia Z., Wang L., Tan T. (2022). Focal and efficient IOU loss for accurate bounding box regression. Neurocomputing.

